# Suggestions on the Treatment of Cholera Patients, Addressed to the Parochial Boards Jointly by a Committee of the Royal College of Physicians and the Royal College of Surgeons of Edinburgh, and Dr. Sutherland, the Commissioner of the General Board of Health

**Published:** 1849-09

**Authors:** 


*Suggestions on the treatment of Cholera Patients, addressed to the Parochial Boards jointly by a committee of the Royal College of Physicians and the Royal College of Surgeons of Edinburgh, and Dr. Sutherland, the Commissioner of the General Board of Health.*—I. At the district dispensaries there should be kept, not only the medicines, but the other materials requisite for the treatment of the disease, in the houses of the poor,—such as straw-mattresses, blankets, vessels for heating sand or salt, spirits of turpentine, and cloths for applying it, mustard for cataplasms, coals and wood for firing; but these are to be given out only on the orders of medical men who have seen the patients. There should also be the necessary messengers, materials for fumigation, and the means of conveyance to hospital.

II. On an application to one of these dispensaries from a patient reported to have diarrhœa, the attendant will proceed thus:—

1. He will issue directly twelve of the pills containing opium, hereinafter specified, with directions to give two immediately, and repeat them every three hours while the diarrhœa lasts; but if there be along with it vomiting or cramps, every hour while these symptoms last, until the medical man arrives.—2. He will give directions for applying external warmth by all available means—blankets, hot bricks, hot sand or salt, turpentine, or mustard poultices, on the abdomen and extremities, bottles of hot water laid alongside the patient, frictions with hot flannels, and as warm covering as possible. 3. He will direct that the patient drink nothing for a quarter of an hour after each dose of the pills, but that at that interval after each dose, he take a table-spoonful of spirits with hot water, or two table-spoonfuls of spiced wine; and if his skin is felt to be cold and damp, repeat this every half-hour.

*N. B.*—In the case of children who are from 10 to 14 years of age, he will be careful to direct half the quantities both of opiates and spirits, and in the younger children proportionally smaller doses. 4. He will give the address of the medical man attached to the district where the patient is, and direct that he be immediately informed of the case, and, if necessary, send a messenger to inform him.

The following pills may be kept constantly at each station, and the medical officers may leave general directions as to the selection of one or other of these in the first issues to the patients :—

℞ Acet. Plumbi, ʒss. ; Opii, gr. xij. ; Conserv. Ros., q. s. Ft. pilulæ xvi. Sign. Lead and Opium Pills.

℞ Tannini, ʒss. ; Opii, gr. xij. ; Pulv. Capsici, gr. xvj. ; Conserv. Ros., q. s. Ft. pilulæ xvi. Sign. Astringent Pills with Opium.

℞ Calomelanos, ʒss. ; Opii, gr. xij. ; Pulv. Capsici, gr. xvj. ; Conserv. Ros., q. s. Ft. pilulæ xvi. Sign. Calomel and Opium Pills.

The doses of all these should be as above directed. Along with these, in the early stage of the disease, and when the skin is cold and damp, such a stimulating mixture as the following, besides the wine and spirits, may be used :—

℞ Ætheris Sulph. ; Spirit. Ammoniæ Aromat. ana ℥ss. ; Tincturæ Cinnamon. Comp. ℥j. (*Misce.*) Sign. Two tea-spoonsful to be taken every half-hour or hour.

III. The medical officers should be reminded of the paramount importance in this disease of early and assiduous treatment and careful watching of the patients, by themselves or trustworthy assistants, *in the early stage:* the object being, if possible, to prevent the patient from falling into a state of collapse, or if he should, to bring on reaction as speedily as possible.

Concurrent testimony is in favour of opium, as the most powerful remedy, provided it be given in *full* and *repeated* doses within the first 12 hours—at farthest within the first 24 hours from the attack—if possible before there is collapse, certainly before there is the tendency to stupor, which is to be expected after the collapse.

When reaction has taken place, and the tendency to stupor shown itself, the farther use of the opium and astringents requires much caution, and the case must be treated as one of febrile disease, particular attention being paid to the quantity and quality of urine passed.

IV. When removal of the patient to hospital is thought necessary, it should be effected in the recumbent posture ; and the litter employed should be so constructed and managed, as to secure, as far as possible, protection and warmth during the removal. Litters of this kind are kept at the cholera hospital, Surgeon Square, and at the Royal Infirmary.

V. When the first patient affected in a house, (particularly if crowded, dirty, and inhabited by destitute people,) has been removed or has died, or in the case where some of the members of the family are of no use for the assistance of the patient, the medical officer will consider the advantage of removing the remaining or less useful members of the family—and when the locality is damp or ill-aired, or the cases have occurred in rapid succession, some of the neighbors—without delay into one of the houses of refuge prepared for their reception: experience having shewn that when this measure has been promptly adopted, successions of fatal cases in the same family, such as have already occurred in at least eight of the places where the disease has recently appeared in Edinburgh, have very generally been averted. If this measure is assented to by the family, he will give immediate notice to the officers of police on his return of the case, that the house may be taken charge of by the police, and thoroughly cleansed. Some objection to this measure on the part of the affected families may always be expected at first, but a little explanation, and a little experience of its effects, will very generally surmount the difficulty.—*London Medical Gazette.*

